# Oral infection with the *Salmonella enterica *serovar Gallinarum 9R attenuated live vaccine as a model to characterise immunity to fowl typhoid in the chicken

**DOI:** 10.1186/1746-6148-1-2

**Published:** 2005-09-12

**Authors:** Paul Wigley, Scott Hulme, Claire Powers, Richard Beal, Adrian Smith, Paul Barrow

**Affiliations:** 1 Department of Veterinary Pathology, University of Liverpool, Leahurst, Neston, CH64 7TE, Merseyside, UK; 2 Institute for Animal Health, Compton Laboratory, Compton, Newbury, UK, RG20 7NN, Berkshire, UK

## Abstract

**Background:**

*Salmonella *enterica serovar Gallinarum (*S*. Gallinarum) is the causative agent of fowl typhoid, a severe systemic disease of chickens that results in high mortality amongst infected flocks. Due to its virulence, the immune response to *S*. Gallinarum is poorly characterised. In this study we have utilised infection by the live attenuated S. Gallinarum 9R vaccine strain in inbred chickens to characterise humoral, cellular and cytokine responses to systemic salmonellosis.

**Results:**

Infection with 9R results in a mild systemic infection. Bacterial clearance at three weeks post infection coincides with increases in circulating anti-*Salmonella *antibodies, increased T cell proliferation to *Salmonella *challenge and increased expression of interferon gamma. These responses peak at four weeks post infection, then decline. Only modest increases of expression of the pro-inflammatory cytokine interleukin-1β were detected early in the infection.

**Conclusion:**

Infection of chickens with the 9R vaccine strain induces a mild form of systemic salmonellosis. This induces both cellular and humoral immune responses, which peak soon after bacterial clearance. Unlike enteric-associated *Salmonella *infections the immune response is not prolonged, reflecting the absence of persistence of *Salmonella *in the gastrointestinal tract. The findings here indicate that the use of the *S*. Gallinarum 9R vaccine strain is an effective model to study immunity to systemic salmonellosis in the chicken and may be employed in further studies to determine which components of the immune response are needed for protection.

## Background

*Salmonella enterica *serovar Gallinarum (*S*. Gallinarum) is the causative agent of fowl typhoid, a severe systemic disease of chickens and other galliforme birds [1]. *S*. Gallinarum is a non-motile Gram negative rod and along with the closely related *Salmonella enterica *serovar Pullorum is host-specific for poultry, but rarely, if ever, presents a risk of zoonotic transmission to man. Infection in chickens may occur at all ages and is typified by severe hepatosplenomegaly accompanied by characteristic liver 'bronzing', anaemia and septicaemia [1]. *S*. Gallinarum is primarily associated with the mononuclear phagocyte system and resides primarily within macrophages in the liver and spleen [2,3]. It is only found in the gastrointestinal tract early in the infection, usually through faecal-oral transmission, and in the end stage of fowl typhoid where bacteria are shed into the intestines leading to substantial haemorrhaging of the intestinal wall [3]. Infection leads to high rates of morbidity and mortality with a recent study describing a mortality rate in excess of 60% in experimentally infected outbred chickens [4]. Although control programmes including vaccination have largely controlled the disease in Europe and North America, it remains of high economic importance to developing poultry industries in Asia and South America.

The high mortality and morbidity rates associated with *S*. Gallinarum make effective study of immunity to infection difficult to achieve. Although studies in inbred genetically resistant and susceptible chickens have demonstrated the role of the innate immune system to some extent [3], the role of the adaptive immune response and in particular cellular responses has not been described other than a few serological studies [1,2,5]. In order to further characterise the immune response to *S*. Gallinarum, we utilised an infection model with the live attenuated fowl typhoid vaccine 9R. The 9R vaccine strain developed in the 1950s, has a 'semi-rough' lipopolysaccharide structure, but the nature of its attenuation is not known [6,7]. Although highly attenuated compared to its parental strain *S*. Gallinarum 9, the 9R vaccine strain still results in systemic disease with pathology in the liver and spleen, and bacterial persistence for several weeks at these sites [5]. Therefore although the vaccine strain does not cause significant mortality, it causes a mild form of systemic salmonellosis. This allows more detailed study of the immune response to be undertaken without using high numbers of animals needed to determine immune responses associated with clearance due to high mortality rates.

In this study we have determined both humoral and cellular immune responses to systemic salmonellosis in an inbred chicken line, and investigate expression of two key cytokines. Line 7_2 _is derived from White Leghorn Chickens and is moderately susceptible to systemic salmonellosis [3]. Previous studies have determined both cellular and humoral responses to *Salmonella enterica *serovar Typhimurium in the chicken [8,9]. Although *S*. Typhimurium is mainly associated with the gastrointestinal tract in chickens, it causes a transient primary systemic infection. Control of this systemic infection appears to be dependent on cell-mediated immunity as clearance of bacteria from the spleen and liver coincides with the height of T-cell proliferative activity and expression of the T helper 1(Th1)-type cytokine interferon-γ (IFN-γ) [8,9]. Infection with *S*. Typhimurium also results in specific IgG, IgM and IgA antibody responses [8], as does vaccination with killed *Salmonella *vaccines [10]. Infection with either *Salmonella *serovar Typhimurium or serovar Enteritidis leads to prolonged high titres of specific antibody, probably as a consequence of the prolonged persistence of these serovars in the gastrointestinal tract, a phenomenon that is not found with *S*. Gallinarum [9,11-13] Whilst it is clear specific antibody protects against secondary systemic infection [10], its role in clearance of primary infection is less clear. Following bursectomy, depletion of the Bursa of Fabricius the primary lymphoid organ associated with B lymphocyte development in the chicken, [14,15], conflicting results have been found on the role of antibody in *Salmonella *clearance. Therefore the role of antibody is not yet clearly defined in primary avian *Salmonella *infection.

In this study we have initiated investigation of immunity to systemic infection with *S*. Gallinarum. We have determined both the specific anti-*S*. Gallinarum antibody (IgG and IgM) response, the cellular response and expression of the key cytokines IFN-γ and Interleukin-1β (IL-1β) following oral infection of inbred chickens with *S*. Gallinarum 9R.

## Results

### The *S*. Gallinarum 9R vaccine strain causes a low level systemic infection

Following infection *S*. Gallinarum 9R was detected in the spleen and liver at one and two weeks post infection (Table 1), though no bacteria were detected by three weeks post infection or at any subsequent time point. At both one and two weeks post infection inflammatory signs including mild to moderate hepatosplenomegaly were detected in infected birds. This level of pathology is consistent with previous studies of the 9R strain [5].

**Table 1:** Counts of *S*. Gallinarum 9R vaccine strain from tissues following oral inoculation of three-week old Line 7_2 _chickens (±SEM)

**Tissue**	**Weeks post infection**					
	1		2		3	
	
	Mean count Log_10 _cfu/g	Number positive	Mean count Log_10 _cfu/g	Number positive	Mean count Log_10 _cfu/g	Number positive

Liver	1.84 ± 0.37	5/5	1.43 ± 0.29	5/5	<1	0/5
Spleen	1.39 ± 0.57	4/5	< 1	3/5*	<1	0/5

*detected only after enrichment in selenite broth

### Cellular and humoral responses to experimental infection with 9R

**Figure 1:**
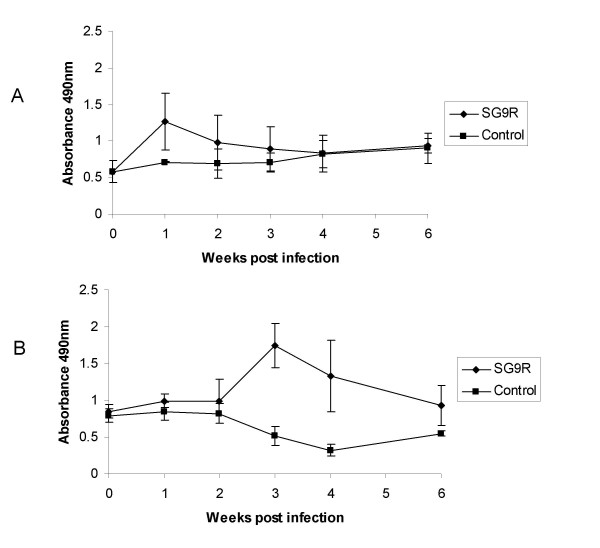
**Antibody responses to *S*. Gallinarum 9R**. Serum antibody responses to S. Gallinarum lysate antigen in *S*. Gallinarum 9R infected and control Line 7_2 _chickens infected orally at three weeks of age as determined by ELISA (±SEM). (A) IgM response (serum diluted 1:50) and (B) IgG response (serum diluted 1:200)

**Figure 2:**
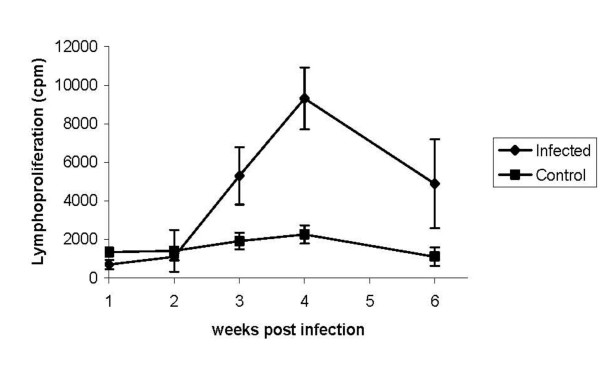
**Antigen-specific T lymphocyte proliferation to *Salmonella***. Antigen-specific proliferation of splenic lymphocytes from Line 7_2 _chickens infected with *S*. Gallinarum 9R or infected controls (±SEM). Proliferation was determined by the uptake of tritiated thymidine measured 48 h after culture of cells with soluble *S*. Gallinarum antigen. Differences between control and infected groups were analysed by ANOVA indicating significantly increased proliferation in infected over control birds at 3 and 4 weeks post infection (P < 0.05)

IgM antibody responses were detected at one week post infection, and subsequently decreased (Figure 1a), whereas IgG responses reached a peak three weeks post infection and subsequently declined (Figure 1b). Cellular responses followed a similar pattern to IgG production with significantly higher levels of T-cell proliferation (P < 0.05) to *Salmonella *antigen in the infected over control groups found at three-to-four weeks post infection (Figure 2). These findings indicate that oral infection with 9R elicits both humoral and cellular immune responses that coincide with clearance of the bacterium from the liver and spleen.

### Cytokine expression following *S*. Gallinarum 9R infection

**Figure 3:**
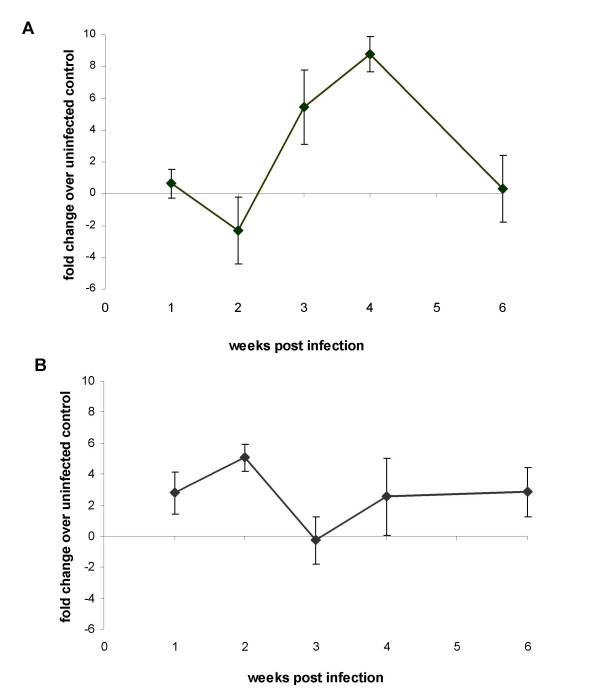
**Expression of IFN-γ and IL1-β following infection with *S*. Gallinarum 9R**. Expression of the cytokines IFN-γ (A) and IL1-β (B) in the spleen of Line 72 chickens infected with *S*. Gallinarum 9R (±SEM). Expression was determined by quantitative reverse-transcription real-time PCR. Data are displayed as the mean fold-change in expression in infected birds (n = 5) in comparison to uninfected controls (n = 5) at the same time point. Differences between control and infected groups were analysed by ANOVA indicating significantly increased expression of IFN-γ in infected over control birds at 3 and 4 weeks post infection (P < 0.05).

Expression of the Th1-type cytokine IFN-γ followed a similar pattern to that of T-cell proliferation with infected birds showing increased expression over the controls at three weeks (P = 0.03) and four weeks (P < 0.01) post infection with up to eight-fold increases in expression found (Figure 3). The increase is consistent with T-lymphocyte activation, particularly Th1 responses. Only modest increases of expression of the pro-inflammatory cytokine IL-1β were found following infection, though significantly greater in infected birds at two weeks post infection (P = 0.02). This increase corresponds to presence of bacteria in the spleen and mild hepatosplenomegaly in the infected group. Previous studies have suggested that presence of high bacterial numbers in the spleen induces high expression levels of IL-1β [9]. However in this study relatively limited inflammation was found and it seems likely that 9R induces only a limited pro-inflammatory response.

## Discussion

The data presented here indicate that systemic Salmonella infection of the chicken induces both cellular and humoral responses in the chicken. Both responses peak at three to four weeks post infection, a point that coincides with bacterial clearance. Previous studies of immune responses in avian salmonellosis have concentrated on virulent or attenuated strains of *S*. Typhimurium and *S*. Enteritidis [9,16-19]. The responses found in these studies also demonstrated both humoral and cellular responses that peak at similar times to those described here, though in contrast to this study, these infections elicit responses that remain considerably higher than in this study, where responses declined rapidly after four weeks post infection. Both *S*. Typhimurium and *S*. Enteritidis may persist in the gastrointestinal tract for many weeks, whereas *S*. Gallinarum infection generally results either in the mortality of susceptible birds or bacterial clearance in resistant birds within three to four weeks of initial infection, although occasionally persistent infection occurs [3,20]. It seems that bacterial persistence in the gastrointestinal tract maintains a more prolonged immune response. In addition persistent, low-level, systemic infection of chickens by *Salmonella *enterica serovar Pullorum leads to prolonged high titre antibody responses and T lymphocyte proliferation [21].

Although the duration of the primary immune response is shorter in *S*. Gallinarum infection, as with the early stages of S. Typhimurium infection in the chicken, clearance from the spleen and liver coincides with increased T lymphocyte proliferation and expression of IFN-γ [9]. Comparative infection studies of *Salmonella-*resistant naked neck chicken breed and commercial layers have also shown correlation between the cellular immune response and protection against fowl typhoid [22]. In many ways the biology of *S*. Gallinarum infection is more akin to S. Typhimurium infection of the mouse than the chicken. In common with *S*. Gallinarum in the chicken, murine infection with S. Typhimurium results in a severe systemic 'typhoid-like' infection. The development of the murine immune response to S. Typhimurium has been well characterised and has recently been reviewed [23]. In the early stages of both murine and avian systemic salmonellosis, bacterial numbers are initially controlled through the innate immune system, and in particular through the generation of reactive oxygen intermediates [3,23]. In the mouse initiation of the adaptive immunity relies on the action of a number of cytokines including IFN-γ, Interleukin-12 (IL-12), Interluekin-18 (IL-18) and tumour necrosis factor-α. Production of IL-12 and IL-18, primarily by macrophages leads to expression of IFN-γ by natural killer cells and T lymphocytes, which in turn leads to increased macrophage activation. These, primarily Th1, cytokine responses lead to the development of CD4^+ ^T-cell responses that lead to clearance of *Salmonella *from the tissues. The response to *S*. Galllinarum 9R mirrors this, with an increase in IFN-γ expression correlating to increased T-cell proliferation and clearance of the vaccine strain from the tissues. Antibody responses, initially IgM followed by IgG are produced to 9R in a classical primary response. These also correlate to the clearance of *Salmonella*. The relative roles of humoral and cellular immunity to clearance are not yet known, though as *S*. Gallinarum are believed to survive and multiply within macrophages [2-4], it would appear to be more likely that Th1-mediated cellular responses are more important in clearance. Further functional studies will be used to determine the relative roles of cellular and humoral responses in protection.

The infection model used in this study is a useful tool in studying the immune response to S. Gallinarum, but without the high mortality or morbidity rates found with virulent strains, even in genetically resistant birds. Similar attenuated vaccines strains have been used extensively in the S. Typhimurium murine model [23] and also to characterise the immune responses to *Salmonella *infection in cattle [24]. The data presented here indicates that an immune response consistent with that seen in other animal models of systemic salmonellosis is elicited following oral infection with the 9R vaccine strain. This represents a valuable model to study immunity to fowl typhoid in the chicken. The studies presented here indicate that infection generates both humoral and a Th1-mediated cellular response. Further studies with this model will allow the determination of which components of the response are protective, aiding future rational avian *Salmonella *vaccine design.

## Conclusion

The *S*. Gallinarum 9R vaccine strain is a suitable model to study the immune response to systemic salmonellosis in the chicken. Infection with 9R induces both antibody and Th1-type T cell responses that are associated with bacterial clearance.

## Methods

### Experimental animals

Specific pathogen-free (SPF) Line 7_2 _inbred White Leghorn chickens were obtained form the Poultry Production Unit, Institute for Animal Health, Compton, UK. Birds were reared on wire cages initially at an ambient temperature of 30°C then at 21°C from three weeks old. Birds were given *ad libitum *access to water and a vegetable protein based diet (SDS, Witham, Essex, UK). All experimental work involving animals was performed under the conditions of a Home Office project licence and of the local ethics committee meeting the requirements of UK legislation.

### Bacterial strains

The *S*. Gallinarum 9R vaccine strain [6] was cultured in Luria Bertani (LB) broth (Difco, Becton-Dickinson Labware, Cowley, Oxford, UK) at 37°C in an orbital shaking incubator at 150 rpm from stocks held at -70°C in LB broth supplemented with 30% glycerol.

### Production of *Salmonella *lysate antigen

A soluble protein antigen lysate preparation was prepared from *S*. Gallinarum 9, the virulent strain from which the 9R vaccine was produced, as previously described [9]. The antigen preparation was subsequently used for ELISA and T-cell proliferation assays

### Experimental infection with the 9R vaccine strain

Fifty 3 week-old Line 7_2 _chickens were divided into two groups of equal size and housed separately as described above. Prior to infection five birds from each group were bled from the wing vein to obtain serum. The birds of one group were then infected orally with 10^8 ^CFU of the *S*. Gallinarum 9R vaccine strain in a volume of 0.3 ml of LB broth. The second group remained uninfected as controls. At 1, 2, 3, 4 and 6 weeks post infection, five birds from each group were killed for post mortem analysis. At each time point samples of spleen and liver were taken aseptically for bacteriological analysis. A section of splenic tissue was taken into RPMI1640 containing 100 U/ml penicillin, 1 μg/ml streptomycin and 5% bovine serum to isolate splenocytes for T-cell proliferation assays. A small sample of splenic tissue was also obtained for isolation of RNA using RNAlater (Qiagen, Crawley, UK) to protect against any degradation. Birds were also bled by cardiac puncture to obtain serum. For bacteriological analysis samples were homogenised in sterile phosphate buffered saline (PBS) using Griffith's tube homogenisers, then serially diluted in PBS and plated onto Brilliant Green Agar (Difco, Becton-Dickinson Labware). Plates were then incubated at 37°C for 24 h, then the bacterial count determined. Samples were also enriched by adding an equal volume of double strength selenite broth, followed by overnight incubation at 37°C. Enriched samples were plated onto Brilliant Green Agar and incubated as described above. Growth was then recorded as *Salmonella *positive or negative.

### Measurement of anti-*Salmonella *antibody responses by ELISA

Anti-*Salmonella *IgG and IgM responses were determined by ELISA on pre-infection and post mortem serum samples using plates coated with *S*. Gallinarum lysate antigen as described previously for S. Pullorum and *S*. Typhimurium [9,25].

### T-cell proliferation assay

Single cell suspensions of splenocytes were prepared from post mortem samples by passing splenic tissue through Falcon cell strainers (Life Technologies, Paisley, UK) in RPMI1640 containing 100 U/ml penicillin, 1 μg/ml streptomycin and 5% bovine serum. The majority of erythrocytes were removed by centrifugation at 35 × *g *for 10 minutes. Cell proliferation to *S*. Gallinarum antigen was determined by uptake of tritiated thymidine as described previously [9].

### Quantitative analysis of cytokine mRNA expression

Total RNA was isolated from RNAlater-protected samples using RNeasy min kits (Qiagen, Crawley, UK) following manufacturer's protocols. Levels of expression of the cytokines IL-1β and IFN-γ were determined by real-time reverse transcription-polymerase chain reaction (RT-PCR) using the ABI Prism 7700 Sequence Detection System (TaqMan^®^; PE Applied Biosystems, Warrington, UK) as previously described [9,26-28]. Values for expression of mRNA were corrected against the expression of 28S rRNA as a 'housekeeping' gene.

### Statistical analysis

Statistical analysis was performed either using Microsoft Excel or Minitab for Windows. Comparison between infected and control groups was made by ANOVA. Values of P < 0.05 were taken as significant.

## Authors' contributions

PW conceived the experimental outline, conducted the *in vivo *experiments, cell proliferation assays, analysed the data and co-wrote the manuscript, SH performed the cytokine expression and assisted with the bacteriology. CP assisted with *in vivo *experiments and performed the ELISA assays, RB developed and assisted with the proliferation assays, AS helped in experimental design and preparation of the manuscript, PB co-designed the experiments and co-wrote the manuscript.

## References

[B1] Shivaprasad HL (2000). Fowl typhoid and pullorum disease. Rev Sci Tech.

[B2] Barrow PA, Huggins MB, Lovell MA (1994). Host specificity of Salmonella infection in chickens and mice is expressed in vivo primarily at the level of the reticuloendothelial system. Infect Immun.

[B3] Wigley P, Hulme SD, Bumstead N, Barrow PA (2002). In vivo and in vitro studies of genetic resistance to systemic salmonellosis in the chicken encoded by the SAL1 locus. Microbes Infect.

[B4] Jones MA, Wigley P, Page KL, Hulme SD, Barrow PA (2001). Salmonella enterica serovar Gallinarum requires the Salmonella pathogenicity island 2 type III secretion system but not the Salmonella pathogenicity island 1 type III secretion system for virulence in chickens. Infect Immun.

[B5] Silva EN, Snoeyenbos GH, Weinack OM, Smyser CF (1981). Studies on the use of 9R strain of Salmonella gallinarum as a vaccine in chickens. Avian Dis.

[B6] Smith HW (1956). The use of live vaccines in experimental Salmonella gallinarum infection in chickens with observations on their interference effect. J Hyg (Lond).

[B7] Smith HW (1956). The immunity to Salmonella gallinarum infection in chickens produced by live cultures of members of the Salmonella genus. J Hyg (Lond).

[B8] Beal RKWPPCHSDBPASAL (2004). Age at primary infection with Salmonella enterica serovar Typhimurium in the chicken influences persistence of infection and subsequent immunity to re-challenge. Vet Immunol Immunopathol.

[B9] Beal RK, Powers C, Wigley P, Barrow PA, Smith AL (2004). Temporal dynamics of the cellular, humoral and cytokine responses in chickens during primary and secondary infection with Salmonella enterica serovar Typhimurium. Avian Pathol.

[B10] Woodward MJ, Gettinby G, Breslin MF, Corkish JD, Houghton S (2002). The efficacy of Salenvac, a Salmonella enterica subsp. Enterica serotype Enteritidis iron-restricted bacterin vaccine, in laying chickens. Avian Pathol.

[B11] Barrow P (1991). Serological analysis for antibodies to S enteritidis. Vet Rec.

[B12] Brito JR, Hinton M, Stokes CR, Pearson GR (1993). The humoral and cell mediated immune response of young chicks to Salmonella typhimurium and S. Kedougou. Br Vet J.

[B13] Cooper GL, Venables LM, Woodward MJ, Hormaeche CE (1994). Vaccination of chickens with strain CVL30, a genetically defined Salmonella enteritidis aroA live oral vaccine candidate. Infect Immun.

[B14] Corrier DE, Elissalde MH, Ziprin RL, DeLoach JR (1991). Effect of immunosuppression with cyclophosphamide, cyclosporin, or dexamethasone on Salmonella colonization of broiler chicks. Avian Dis.

[B15] Desmidt M, Ducatelle R, Mast J, Goddeeris BM, Kaspers B, Haesebrouck F (1998). Role of the humoral immune system in Salmonella enteritidis phage type four infection in chickens. Vet Immunol Immunopathol.

[B16] Babu U, Dalloul RA, Okamura M, Lillehoj HS, Xie H, Raybourne RB, Gaines D, Heckert RA (2004). Salmonella enteritidis clearance and immune responses in chickens following Salmonella vaccination and challenge. Vet Immunol Immunopathol.

[B17] Babu U, Scott M, Myers MJ, Okamura M, Gaines D, Yancy HF, Lillehoj H, Heckert RA, Raybourne RB (2003). Effects of live attenuated and killed Salmonella vaccine on T-lymphocyte mediated immunity in laying hens. Vet Immunol Immunopathol.

[B18] Berndt A, Methner U (2001). Gamma/delta T cell response of chickens after oral administration of attenuated and non-attenuated Salmonella typhimurium strains. Vet Immunol Immunopathol.

[B19] Dueger EL, House JK, Heithoff DM, Mahan MJ (2001). Salmonella DNA adenine methylase mutants elicit protective immune responses to homologous and heterologous serovars in chickens. Infect Immun.

[B20] Wigley P (2004). Genetic resistance to Salmonella infection in domestic animals. Res Vet Sci.

[B21] Wigley P, Hulme SD, Powers C, Beal RK, Berchieri AJ, Smith A, Barrow P (2005). Infection of the Reproductive Tract and Eggs with Salmonella enterica Serovar Pullorum in the Chicken Is Associated with Suppression of Cellular Immunity at Sexual Maturity. Infect Immun.

[B22] Alvarez MT, Ledesma N, Tellez G, Molinari JL, Tato P (2003). Comparison of the immune responses against Salmonella enterica serovar Gallinarum infection between naked neck chickens and a commercial chicken line. Avian Pathol.

[B23] Mastroeni P, Menager N (2003). Development of acquired immunity to Salmonella. J Med Microbiol.

[B24] Villarreal-Ramos B, Manser J, Collins RA, Dougan G, Chatfield SN, Howard C (1998). Immune responses in calves immunised orally or subcutaneously with a live Salmonella typhimurium aro vaccine.. Vaccine.

[B25] Wigley P, Berchieri AJ, Page KL, Smith AL, Barrow PA (2001). Salmonella enterica serovar Pullorum persists in splenic macrophages and in the reproductive tract during persistent, disease-free carriage in chickens. Infect Immun.

[B26] Kaiser P, Rothwell L, Galyov EE, Barrow PA, Burnside J, Wigley P (2000). Differential cytokine expression in avian cells in response to invasion by Salmonella typhimurium, Salmonella enteritidis and Salmonella gallinarum. Microbiology.

[B27] Kaiser P, Underwood G, Davison F (2003). Differential cytokine responses following Marek's disease virus infection of chickens differing in resistance to Marek's disease. J Virol.

[B28] Withanage GS, Kaiser P, Wigley P, Powers C, Mastroeni P, Brooks H, Barrow P, Smith A, Maskell D, McConnell I (2004). Rapid expression of chemokines and proinflammatory cytokines in newly hatched chickens infected with Salmonella enterica serovar typhimurium. Infect Immun.

